# ΜicroRNA (miRNA) Variants in Male Infertility: Insights from Whole-Genome Sequencing

**DOI:** 10.3390/genes15111393

**Published:** 2024-10-29

**Authors:** Maria-Anna Kyrgiafini, Veselin Veselinov Vasilev, Alexia Chatziparasidou, Zissis Mamuris

**Affiliations:** 1 Laboratory of Genetics, Comparative and Evolutionary Biology, Department of Biochemistry and Biotechnology, University of Thessaly, Viopolis, Mezourlo, 41500 Larissa, Greece; 2 Embryolab IVF Unit, St. 173-175 Ethnikis Antistaseos, Kalamaria, 55134 Thessaloniki, Greece

**Keywords:** male infertility, microRNA (miRNA), variant, noncoding RNA (ncRNA), biomarker

## Abstract

Background/Objectives: Male infertility is a complex condition with various underlying genetic factors. microRNAs (miRNAs) play a crucial role in gene regulation, and their disruption can significantly impact fertility. This study aimed to identify variants within miRNA genes and elucidate their impact on male infertility. Methods: Whole genome sequencing was performed on blood samples from men with asthenozoospermia, oligozoospermia, and teratozoospermia, compared to normozoospermic controls. The analysis revealed a significant number of unique variants in each infertile group. We subsequently focused on variants in miRNA regions, followed by an in silico analysis to investigate the role of the identified variants and miRNAs in male infertility. Results: Focused analysis on miRNA genes identified 19 exclusive variants in teratozoospermic men, 24 in asthenozoospermic, and 27 in oligozoospermic, all mapping to pre-miRNAs or mature miRNAs. Functional analyses using Gene Ontology (GO) and KEGG pathways highlighted key biological processes and pathways disrupted by these variants and miRNA–mRNA interactions, including transcription regulation, signaling, and cancer-related pathways. Furthermore, six variants (rs17797090, rs1844035, rs7210937, rs451887, rs12233076, and rs6787734) were common across the infertile groups, suggesting their importance in male infertility or their potential as biomarkers. Common variants were also validated in another clinically relevant group of men. Some miRNAs with identified variants, such as hsa-miR-449b and hsa-miR-296, have been previously implicated in male infertility and exhibit differential expression between fertile and infertile men, according to the literature, too. Conclusion: These results provide new insights into the genetic basis of male infertility and open avenues for future research and therapeutic interventions.

## 1. Introduction

Infertility, defined as the inability to achieve pregnancy after one year of regular unprotected sexual intercourse, according to the World Health Organization (WHO), is a multifactorial condition with significant implications for reproductive health. It is estimated that at least 180 million couples worldwide are facing infertility problems (WHO), and, in many cases, the male factor plays an important role. More specifically, males are solely responsible for about 20% of cases and contribute to another 30% of all infertility cases where male and female causes co-exist [1]. Thus, overall, the male factor substantially contributes to about 50% of all cases of infertility [1]. While the etiology of male infertility is diverse, encompassing genetic, environmental, and lifestyle factors, it often involves abnormalities in sperm production, function, or delivery [1,2]. Common sperm abnormalities include asthenozoospermia, teratozoospermia, and oligozoospermia. Asthenozoospermia refers to the condition in which spermatozoa demonstrate reduced motility; teratozoospermia involves the presence of abnormal sperm morphology; and oligozoospermia is characterized by a low sperm count in the ejaculate [2]. Male infertility affects around 7% of the global male population [3], with prevalence rates varying between different regions and populations [1,4]. Regarding specific subtypes of male infertility, data on their prevalence are extremely limited to specific regions and not worldwide. However, it is estimated that complete asthenozoospermia, showing 100% immotile spermatozoa, is found with a frequency of 1 in 5000 men [5]. For the other subtypes, there is no estimation.

Therefore, gaining a better understanding of the underlying mechanisms of male infertility is crucial for the development of effective diagnostic tools and therapeutic interventions to address this widespread reproductive health problem.

In recent years, noncoding RNAs (ncRNAs), especially microRNAs (miRNAs), have emerged as critical regulators of gene expression and essential players in various physiological processes. Consequently, their dysregulation has been associated with the development of several diseases [6,7,8]. miRNA genes are found in intragenic and intergenic regions and are transcribed by RNA polymerase II and III [9]. This transcription yields a hairpin-like molecule, which is subsequently processed by the RNase III enzyme Drosha to produce a precursor microRNA (pre-microRNA, pre-miRNA) of approximately 70 nucleotides in length [9]. Exportin-5 facilitates the transport of this precursor to the cytoplasm, where it undergoes further cleavage by the Ago2/Dicer complex, generating small mature double strands of microRNA [9]. Typically, the passenger strand is degraded, leaving the guide strand to form the RNA-induced silencing complex (miRISC) [9]. Through this complex, microRNAs perform their gene regulatory functions by binding to specific messenger RNA (mRNA) targets [9]. Studies show that a single microRNA can target multiple mRNAs, while conversely, one mRNA may possess binding sites for numerous microRNAs [10,11,12,13]. This mechanism enables miRNAs to exert their function and affect gene regulation. miRNAs have a crucial role in processes such as spermatogenesis and sperm function [14,15]. Therefore, since miRNAs have been found in the testis, epididymis, sperm cells, and seminal plasma, the dysregulation of miRNA expression has been implicated in the pathogenesis of male infertility, contributing to abnormalities in sperm production, maturation, and fertilization [16].

Although the role of miRNAs in male infertility is widely acknowledged, our understanding of genetic variations within miRNA genes and their potential implications for male infertility remains limited. Specifically, very little is known about the genetic variations of miRNA genes and how they may affect the functioning of miRNAs and, consequently, the development of male infertility. The investigation of these genetic variants is crucial, as single nucleotide polymorphisms (SNPs) within miRNA genes have the potential to alter the binding affinity of miRNAs to their target mRNAs. Furthermore, variants within miRNA genes can disrupt miRNA biogenesis or stability, thus impairing the processing of primary miRNA transcripts into mature, functional miRNAs [17,18]. These alterations can lead to dysregulated gene expression patterns that are essential for spermatogenesis and sperm function, ultimately resulting in male infertility.

Therefore, given the significant knowledge gap in understanding the role of miRNA genes in male infertility, our primary objective is to identify variants within these genes and elucidate their impact on male infertility. To achieve this, we performed whole-genome sequencing (WGS) to identify variants exclusive to infertile men. Then, we focused on examining only variants within miRNA genes, their corresponding targets, and the pathways influenced by these miRNAs, utilizing various tools and analyses. Through this integrated approach, we seek to unravel the intricate interplay between genetic variations in miRNA genes, miRNA–target interactions, and the dysregulated pathways underlying male infertility, providing valuable information for improving its diagnosis and therapy.

## 2. Materials and Methods

### 2.1. Study Participants

This research, conducted in collaboration with the “Embryolab” IVF Unit (Thessaloniki, Greece) as part of the Spermogene research program (Grant number T1E∆K-02787), involved the collection of human blood and sperm samples from volunteers. Before sample collection, all participants provided their informed written consent, and ethical approval for the study was obtained from the Ethics Committee of the University of Thessaly in Volos, Greece.

To gather comprehensive information, volunteers completed a questionnaire concerning their health, medical history, health habits, and other relevant details. Exclusion criteria included patients with varicocele, reproductive tract infections, testicular injuries or pathologies, a history of cryptorchidism, orchitis, or epididymitis, as well as those with specific systemic diseases. Regarding exclusion criteria, it should be noted that extensive genetic testing was performed to exclude males with Y microdeletions, chromosomal abnormalities, or any other genetic causes of infertility and include only samples with idiopathic male infertility. Additionally, we used whole-genome sequencing (WGS) data to ensure that the infertility observed in our study was not due to any other known genetic mutations or variants. Demographic details of the participants are outlined in Table 1.

All volunteers underwent an andrological examination, and semen analysis was conducted on the collected samples. The semen analyses were conducted at the certified andrology laboratory of the Embryolab IVF unit, which follows standardized protocols for evaluating sperm count, motility, morphology, and other relevant factors. It is important to note that sperm samples were obtained through masturbation after abstaining from sexual activity for a minimum period of two to three days. Semen analyses were performed according to the fifth edition of the World Health Organization (WHO) manual for the examination and processing of human semen (https://apps.who.int/iris/handle/10665/44261, accessed on 7 September 2024), which provides criteria for evaluating semen characteristics such as volume, sperm count, motility, and morphology. Based on the reference values of this edition, the individuals were classified as normozoospermic, asthenozoospermic, oligozoospermic, or teratozoospermic. The diagnosis of abnormal sperm parameters was made based on the results of semen analysis. For example, teratozoospermic samples had morphology below the reference values, while all other parameters were normal; oligozoospermic samples had sperm counts below the reference value, etc. It should also be noted that in cases of abnormal findings, it is considered good clinical practice to repeat the semen analysis after a period of time to account for potential variability in sperm parameters. This approach helps ensure that the abnormal result is not due to transient factors, such as illness or temporary lifestyle influences. For the men with abnormal sperm parameters in this study, repeat semen analyses were performed after three to six months to confirm the consistency of the initial findings. Cell vision counting slides (Tek-Event, Sydney, Australia) were used for cell counting, and Nikon Eclipse TS100, E200, and Ts2 microscopes (Minato, Japan) were utilized for observation during semen analysis. Furthermore, based on the questionnaire completed by the volunteers, we ensured the fertility status by including only normozoospermic men with at least one previous pregnancy, while men diagnosed with abnormal sperm parameters reported no history of previous pregnancies.

### 2.2. DNA Extraction and Sample Preparation

Whole blood samples were collected into tubes containing ethylenediaminetetraacetic acid (EDTA). DNA extraction was performed using 200 μL of blood samples and the PureLink Genomic DNA Mini Kit (Invitrogen, Waltham, MA, USA—Catalog number: K182002), following the manufacturer’s guidelines. Quantitative DNA analysis was conducted using the Qubit 2.0 fluorometer and the Qubit dsDNA BR Assay Kit (Invitrogen, Waltham, MA, USA—Catalog number: Q32850). The integrity of the DNA was also evaluated through agarose gel electrophoresis.

Then, to prepare the samples for whole genome sequencing (WGS), we established five separate sequencing pools. DNA from ten individuals with normozoospermia was divided into two pools, with each pool containing DNA from five individuals. A third pool consisted of DNA from five individuals with asthenozoospermia, a fourth pool contained DNA from five individuals with teratozoospermia, and the final pool contained DNA from five oligozoospermic men. The DNA in each pool was mixed to achieve equimolar concentrations, resulting in a uniform final concentration of 100 ng/uL and a total quantity of 2 mg.

### 2.3. Whole Genome Sequencing (WGS) and Data Analysis

Following the sample preparation, whole genome sequencing was performed by Novogene (Cambridge, UK). The genomic DNA, prepared as described earlier, was used to construct libraries for WGS. These libraries underwent normalization and rigorous quality control checks before being sequenced on the Illumina HiSeq 3000 platform (Illumina Inc., San Diego, CA, USA), producing 100 bp paired-end reads and achieving an average coverage of 30×.

The data produced by WGS were subsequently analyzed. Initially, the quality of the generated FASTQ files was assessed using FASTQC [19]. Low-quality reads (with a minimum PHRED score of 30) and adapter sequences were then trimmed using Trimmomatic (v0.39) [20]. Following quality control, the processed reads were aligned to the human reference genome (GRCh37/hg19) sourced from the Ensembl database [21] using the Burrows-Wheeler aligner (BWA) (version 0.7.17) [22]. Duplicate reads resulting from the polymerase chain reaction (PCR) were removed using Picard tools (http://broadinstitute.github.io/picard/, accessed on 7 September 2024), and SAM files were converted to BAM format with SAMtools (v1.19.2) [23]. The BAM files for normozoospermic samples were then merged into a single file for further analysis, also using SAMtools (v1.19.2) [23].

Variant calling was performed using FreeBayes (v1.3.7) [24], with the results being compiled in variant call format (VCF). A comparative analysis of VCF files was then conducted using BCFtools (v1.17) [23] to identify unique genetic variants specific to asthenozoospermic, oligozoospermic, and teratozoospermic conditions. Specifically, VCF files from these three categories were compared with those of normozoospermic men, resulting in three VCF files containing variants unique to each of the infertile groups. These unique variants, not shared between fertile and infertile men, were used for subsequent analyses to explore their potential role in contributing to the pathogenic phenotypes and to provide insights into the molecular mechanisms underlying male infertility. Finally, these variants were annotated using the Variant Effect Predictor (VEP) tool (https://www.ensembl.org/Tools/VEP, accessed on 7 September 2024) [25] provided by the Ensembl database.

### 2.4. Bioinformatics Analysis

After identifying unique variants exclusive to teratozoospermic, asthenozoospermic, or oligozoospermic individuals, only those mapped to miRNA genes, based on the annotation analysis performed, were selected for further analyses as the objective of this study was to explore the role of miRNAs in male infertility. Specifically, various tools and databases were employed to investigate the impact of these variants and the miRNAs affected on male infertility.

Initially, miRNASNP v3 [26] and SNPnexus [27] were utilized to identify whether the unique variants were located within specific pre-miRNAs, mature miRNAs, or seed regions, generating a list of miRNAs potentially involved in male infertility. Subsequently, miRTargetLink 2.0 [28] was employed to identify the target genes of these miRNAs using only validated experimental interactions. To further elucidate the role of these gene targets and uncover deregulated pathways in male infertility associated with exclusive variants, Gene Ontology (GO) enrichment analysis [29,30] and KEGG pathway analysis [31] were performed using ShinyGO 0.77 [32]. It is important to note that for both analyses, statistical significance was determined after correcting for the false discovery rate (FDR) to address multiple comparisons. Specifically, an FDR-adjusted *p*-value threshold of <0.05 was applied. Additionally, only the overlapping gene targets of the listed miRNAs were utilized for both the GO enrichment analysis and the KEGG pathways analysis.

Furthermore, the functional role of the identified variants was examined. SNPnexus [27] was employed to gather information about population genetics and previous associations of variants with diseases or other pathological conditions. Variants potentially possessing functional significance were also identified based on data from the 3DSNP 2.0 [33] and RegulomeDB databases [34]. RegulomeDB integrates data from various sources to discern the regulatory roles of noncoding SNPs. It assigns scores to SNPs, enabling the identification of functional SNPs among a vast pool of variants. Each SNP receives a rank ranging from 1 to 7, with lower values indicating a higher likelihood of possessing a regulatory function [34]. Similarly, the 3DSNP database provides insights into 3D-interacting genes, enhancer states, promoter states, transcription factor binding sites, altered sequence motifs, and conservation. It also assigns a functional score to each SNP, with higher scores signifying a greater likelihood of SNP functionality [33]. Thus, to evaluate the potential regulatory role of variants, only those meeting the criteria of a 3DSNP score > 10 or a RegulomeDB Rank between 1a and 2c were considered significant. It should be noted that for this part of the analysis, only variants identified to be in specific pre-miRNAs, mature miRNAs, or seed regions according to miRNASNP v3 [26] and SNPnexus [27] were used.

### 2.5. Validation of the Identified Variants and Genotyping

Finally, we validated the variants identified as common among infertile men in this study by using another clinically relevant group of men from one of our previous publications [35]. In summary, the study included 365 male participants. After semen analysis was conducted by a certified andrology laboratory (“Embryolab” IVF unit), the samples were divided into control (normozoospermia) and case (abnormal semen parameters) groups. The control group consisted of 280 samples with normal semen parameters (normozoospermia), defined as sperm count > 15 × 10^6^ mL^−1^, total sperm count > 39 × 10^6^, total motility > 40% motile sperm, progressive motility > 32% (Grade a + b) motile sperm, and sperm with normal morphology > 4%. The case group consisted of 85 samples with azoospermia or severe oligozoospermia. Samples were classified as azoospermic or severely oligozoospermic after at least two semen analyses conducted at 2–4-week intervals. As in this study, normozoospermic men reported at least one previous pregnancy, while men diagnosed with abnormal sperm parameters reported no history of previous pregnancies.

Genomic DNA was extracted from the blood samples of the above men, and the concentration and purity of the extracted DNA were carefully assessed. Once prepared, the purified DNA samples were sent for genotyping. Genotyping was performed using the Illumina Infinium^®^ Global Screening Array (Illumina, San Diego, CA, USA). A statistical analysis was then conducted to identify differences in the frequency of the variants identified in the present study between the case and control groups. All the analyses were conducted using the PLINK software v1.07 [36].

## 3. Results

### 3.1. Whole Genome Sequencing—Variant Annotation

Following whole-genome sequencing, data analysis was conducted. More specifically, a comparison between normozoospermic and teratozoospermic individuals was undertaken to identify unique variants present exclusively in one of the two groups. The analysis revealed 617,722 variants specifically observed in teratozoospermic individuals, while 2,342,243 variants were exclusively present in normozoospermic men. These were mapped to 34,603 and 22,022 genes and characterized noncoding regions (such as miRNAs, long noncoding RNA genes, etc.) in normozoospermic and teratozoospermic males, respectively. Similarly, a total of 680,099 variants were exclusively observed in asthenozoospermic individuals, whereas 2,329,803 variants were found only in normozoospermic men. These variants were mapped to 30,362 and 26,019 genes in normozoospermic and asthenozoospermic males, respectively. Finally, 717,374 variants were found in oligozoospermic individuals, while 2,260,073 variants were present exclusively in normozoospermic men. These were mapped to 34,650 and 26,451 genes in normozoospermic and oligozoospermic males, respectively.

Subsequently, as the objective of this study was to identify variants in miRNA genes and elucidate their role in male infertility, we focused only on exclusive variants mapped to these regions. Therefore, annotation using VEP revealed 107 exclusive variants mapped to miRNA regions in teratozoospermic individuals, 89 in asthenozoospermic individuals, and 121 in oligozoospermic individuals. For teratozoospermia, the variants were distributed across chromosomes 1–9, 12–17, 19, 21, 22, X, and Y, with one additional variant found in mitochondrial DNA (mtDNA). Notably, 18 of the identified variants were characterized as novel. In asthenozoospermic men, variants were distributed across all chromosomes except chromosome 18, with two variants also found in mtDNA. Among these, 14 variants were novel, too. Finally, for oligozoospermia, variants were found in all chromosomes except chromosomes 22 and Y, with six variants identified also in mtDNA. Among these, 27 variants were characterized as novel. These results are presented in Table S1.

### 3.2. Variants Within miRNA Regions and miRNAs Affected

For subsequent analysis, we used only variants found within miRNA regions according to miRNASNP v3 [26] and SNPnexus [27]. More specifically, the variants were categorized according to their specific location (pre-miRNAs, mature miRNAs, and seed regions). For teratozoospermia, as presented in Table 2, 19 SNPs were found within pre-miRNA regions, and four of them were also mapped to seed regions. The seed region typically encompasses nucleotides 2–8 from the 5′ end of the mature miRNA sequence. It is highly conserved among different miRNAs and is complementary to sequences within the target messenger RNAs (mRNAs) [37].

Similarly, for asthenozoospermia, as presented in Table 3, 24 SNPs were found within miRNA regions according to miRNASNP v3 [26] and SNPnexus [27]. Among these, three were also mapped to seed regions, and two were located in mature miRNA regions.

Finally, for oligozoospermia, 27 SNPs were identified within miRNA regions according to miRNASNP v3 [26] and SNPnexus [27]. Among these, four were also located in mature miRNAs, and three were in seed regions (Table 4).

Therefore, a comprehensive list of miRNAs affected by the exclusive variants was created for teratozoospermia, asthenozoospermia, and oligozoospermia (Table 2, Table 3 and Table 4).

### 3.3. Investigation of Target Genes of Affected miRNAs

Subsequently, an investigation was carried out to determine the overlap of target genes of the affected miRNAs, aiming to identify pathways that become deregulated in teratozoospermia, asthenozoospermia, and oligozoospermia due to the presence of exclusive variants. Initially, for teratozoospermia, affected miRNAs targeted a total of 2020 common genes, as revealed by miRTargetLink 2.0 [28]. Similarly, for asthenozoospermia, 2730 genes were targeted, and for oligozoospermia, 2832 genes were identified as targets of the affected miRNAs due to exclusive variants. The complete list of these genes as well as their interactions with miRNAs for teratozoospermia, asthenozoospermia, and oligozoospermia is provided in Table S2. It is important to note that both weak and strong validated interactions were selected for constructing miRNA–mRNA interaction networks in all cases.

**Figure 1:**
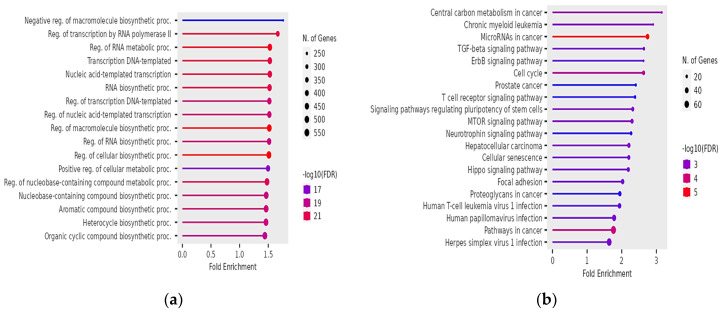
Significant GO biological processes in terms of target genes of affected miRNAs in teratozoospermia (**a**); significant KEGG pathways in terms of target genes of affected miRNAs in teratozoospermia (**b**). The size and color of the dots represent the number of genes and the range of statistical significance, respectively. The y-axis represents the GO terms for biological processes and KEGG pathways terms, respectively, and the x-axis represents the fold enrichment. The *p*-values were corrected for multiple tests using the false discovery rate (FDR).

Then, we used ShinyGO 0.77 [32] for Gene Ontology (GO) and KEGG pathway analyses on the overlap of the above target genes of the miRNAs affected by exclusive variants in teratozoospermic, asthenozoospermic, and oligozoospermic men. For teratozoospermia, the top GO biological processes (GO BP) terms identified were negative regulation of macromolecule biosynthetic processes, regulation of transcription by RNA polymerase II, regulation of RNA metabolic processes, transcription DNA-templated, and nucleic acid-templated transcription (Figure 1a). The key GO Cellular Component (GO CC) term was nuclear inclusion body, while the top GO Molecular Function (GO MF) terms included DNA-binding transcription activator activity, DNA-binding transcription activator activity RNA polymerase-II specific binding, transcription regulatory region nucleic acid binding, and sequence-specific DNA binding (Figures S1 and S2). KEGG pathway analysis also highlighted pathways such as central carbon metabolism in cancer, chronic myeloid leukemia, miRNAs in cancer, TGF-β, and ErbB signaling pathways (Figure 1b).

**Figure 2:**
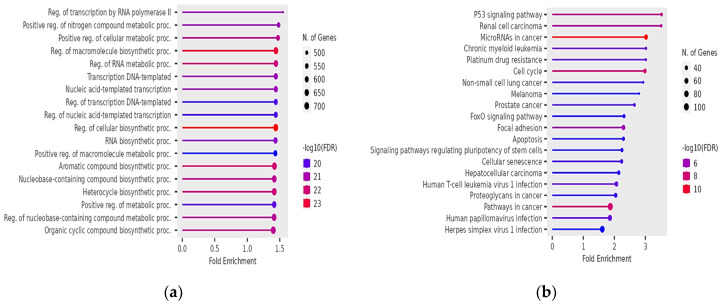
Significant GO biological processes terms of target genes of affected miRNAs in asthenozoospermia (**a**); significant KEGG pathways terms of target genes of affected miRNAs in asthenozoospermia (**b**). The size and color of the dots represent the number of genes and the range of statistical significance, respectively. The y-axis represents the GO terms for biological processes and KEGG pathways terms, respectively, and the x-axis represents the fold enrichment. The *p*-values were corrected for multiple tests using the false discovery rate (FDR).

For asthenozoospermia, the top GO biological processes were regulation of transcription by RNA polymerase II, positive regulation of nitrogen compound metabolic processes, positive regulation of cellular metabolic processes, regulation of macromolecule biosynthetic processes, and regulation of RNA metabolic processes (Figure 2a). Important GO Cellular Components terms included cullin-RING ubiquitin ligase complex, ubiquitin ligase complex, transferase complex, cell leading edge, and transcription regulator complex (Figure S3). Furthermore, among the top GO Molecular Functions terms identified were sequence-specific DNA binding, RNA polymerase II transcription regulatory region sequence-specific DNA binding, sequence-specific double-stranded DNA binding, transcription regulatory region nucleic acid binding, and enzyme binding (Figure S4). Finally, among the top KEGG pathways identified were the p53 signaling pathway, renal cell carcinoma, miRNAs in cancer, chronic myeloid leukemia, platinum drug resistance, and cell cycle (Figure 2b).

**Figure 3:**
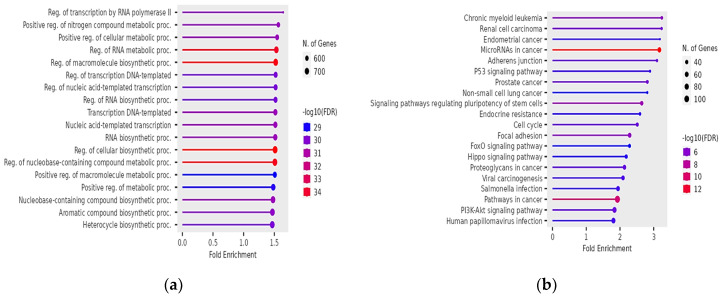
Significant GO biological processes terms of target genes of affected miRNAs in oligozoospermia (**a**); significant KEGG pathways terms of target genes of affected miRNAs in oligozoospermia (**b**). The size and color of the dots represent the number of genes and the range of statistical significance, respectively. The y-axis represents the GO terms for biological processes and KEGG pathways terms, respectively, and the x-axis represents the fold enrichment. The *p*-values were corrected for multiple tests using the false discovery rate (FDR).

Similarly, for oligozoospermia, significant GO biological processes terms identified were regulation of transcription by RNA polymerase II, positive regulation of nitrogen compound metabolic processes, positive regulation of cellular metabolic processes, regulation of RNA metabolic processes, and regulation of macromolecule biosynthetic processes (Figure 3a). For GO CC, the top terms identified were transferase complex, ubiquitin ligase complex, transferase complex transferring phosphorus-containing groups, cell leading edge, and focal adhesion (Figure S5). The top GO MF terms were DNA-binding transcription factor binding, transcription factor binding, sequence-specific DNA binding, cis-regulatory region sequence-specific DNA binding, and transcription regulatory region nucleic acid binding (Figure S6). Finally, the gene targets of affected miRNAs in oligozoospermia were enriched for chronic myeloid leukemia, renal cell carcinoma, endometrial cancer, miRNAs in cancer, adherens junction, and p53 signaling pathway, according to KEGG pathways (Figure 3b).

### 3.4. Investigation of miRNA Variants

In this study, we also conducted a comprehensive evaluation of exclusive variants found only in infertile men and mapped in miRNAs. More specifically, we utilized the 3DSNP 2.0 [33] and RegulomeDB databases [34] to assess their functional significance. Variants with a 3DSNP score exceeding 10 or a RegulomeDB Rank between 1a and 2c were considered to have potentially significant regulatory impact. For teratozoospermia, we identified 8 SNPs as functionally significant (Table 5). Among them, two were considered significant according to both 3DSNP 2.0 and RegulomeDB.

Similarly, in the case of asthenozoospermia, a total of 12 SNPs were identified to have functional significance. Notably, four of these SNPs were found to be significant according to both databases (Table 6).

Finally, 13 SNPs were also identified in oligozoospermia, and three of them were found to be significant in both databases (Table 7). 3DSNP scores for all variants can be found in detail in Table S3, while the RegulomeDB scores are listed in Table S4. Furthermore, according to SNPnexus [27], no significant associations with diseases or other pathological conditions were found for any of the variants.

### 3.5. Common miRNA Variants

Finally, we identified common exclusive variants among infertile men, as shown in Table 8. Specifically, six variants were found across all categories of infertile men (teratozoospermia, asthenozoospermia, oligozoospermia). Additionally, four variants were identified in both teratozoospermia and asthenozoospermia, four in both teratozoospermia and oligozoospermia, and seven in both asthenozoospermia and oligozoospermia.

### 3.6. Validation of Common Variants—Genotyping Results

In this study, we identified by WGS several genetic variants common among infertile men compared to normozoospermic controls (Section 3.5). The validation process, using a clinically relevant group from a previous study [35] further confirmed the consistency of these findings. As observed in Table 9, statistical analysis revealed significant differences in the frequency of eight variants between the two groups (*p*-value < 0.05), suggesting a potential association between these genetic markers in miRNA regions and male infertility. All of the variants exhibited significantly higher frequency in the cases group, which is consistent with the findings of this study. However, it should be noted that some of the variants presented in Table 9 were not identified in the genotyping dataset as the screening array included approximately 700,000 variants across the human genome.

### 3.7. Common miRNA Variants and Differential Expression of miRNAs

In this study, we further explored the biological relevance of the identified common miRNA variants (Table 8) by investigating their expression in male reproductive tissues. A comprehensive literature search was conducted to identify datasets reporting the differential expression of these miRNAs in semen, seminal plasma, or testis tissues. We focused on studies that compared miRNA expression between fertile and infertile men or other relevant comparisons associated with reproduction. As shown in Table 10, six miRNAs are expressed in male reproductive tissues. For each miRNA of interest, we documented the tissue in which it is expressed, the specific comparison (e.g., fertile vs. teratozoospermic patients), and any reported functional roles. This information is presented in Table 10, which highlights the expression patterns and potential regulatory functions of the miRNAs associated with the variants identified in our study.

## 4. Discussion

Male infertility is a complex condition influenced by various genetic, epigenetic, and environmental factors [1]. Recent research has highlighted the critical role of miRNAs in gene regulation. miRNAs function as post-transcriptional regulators of gene expression by binding to target mRNAs and inhibiting their translation or promoting their degradation [48]. Dysregulation of miRNAs can significantly impact numerous biological processes, including those essential for male fertility, such as spermatogenesis [49]. Variants in miRNAs can disrupt these regulatory functions, leading to altered gene expression and subsequent impairment of biological processes [17,18]. However, as little information is available regarding miRNA variants and male infertility, this study aimed to identify variants within miRNA genes and elucidate their impact on male infertility. For this reason, whole genome sequencing was performed on blood samples from men with asthenozoospermia, oligozoospermia, and teratozoospermia and compared to normozoospermic controls. The analysis revealed a significant number of unique variants in each infertile group. Focused analysis on miRNA genes identified 19 exclusive variants in teratozoospermic men, 24 in asthenozoospermic men, and 27 in oligozoospermic men, all mapping to pre-miRNAs or mature miRNAs. The target genes of the affected miRNAs were identified, and KEGG and GO analyses were used to determine deregulated pathways. Furthermore, the functional significance of SNPs was assessed, and common exclusive SNPs were identified. Our findings were further validated using a clinically relevant dataset from a previous publication [35]. The genotyping analysis performed between fertile and infertile men in this additional cohort confirmed the association of the identified genetic variants with male infertility. This validation approach reduces potential assumptions and enhances the generalizability of the results. However, future studies with larger, more diverse cohorts will be essential to confirm these genetic markers and explore their potential role in clinical diagnostics and treatment strategies for male infertility.

### 4.1. miRNAs Affected by Exclusive Variants

Firstly, among the miRNAs identified with exclusive variants, several are implicated in cancer. These include hsa-mir-3652 [50], hsa-mir-618 [51,52], hsa-mir-1269b [53], and hsa-mir-612 [54,55], among others.

In addition, many of these miRNAs are associated with female fertility. For example, hsa-mir-200b has been detected in human endometrial fluid samples and is considered a potential non-invasive biomarker for implantative endometrium [56]. Similarly, hsa-mir-4441 is linked to damage to tubal reproductive functions associated with tubal endometriosis [57], and hsa-mir-4467 is differentially expressed (DE) in exosomes derived from endometrial stromal cells of women with endometriosis-associated infertility [58]. Furthermore, hsa-mir-650, known for its role in cancer, has also been implicated in endometrial receptivity [59]. Additionally, mir-663b levels in human follicular fluid samples are significantly negatively related to viable blastocyst formation [60]. Given these associations, these miRNAs could also potentially influence male infertility and warrant further investigation in future studies.

Importantly, our study identified several variants in miRNAs known to influence male infertility. For example, exclusive variants were detected in hsa-mir-548u in both teratozoospermic and asthenozoospermic samples; this miRNA is differentially expressed in the seminal plasma of patients with Sertoli cell-only syndrome [43]. Additionally, an exclusive variant affecting hsa-mir-612 in oligozoospermic and asthenozoospermic samples was found, and along with *CCL3*, it was listed among the top 10 differentially expressed miRNA target gene pairs in severe oligozoospermia in a study performed by Z. Li et al. (2016) [61]. Another significant finding relates to hsa-mir-296, as hsa-miR-296-5p has shown potential as a biomarker for male infertility [38]. This miRNA is DE in men with unexplained asthenozoospermia [62] and between normozoospermic fertile and infertile individuals [63]. Additionally, hsa-miR-296-5p was found to be downregulated in the human spermatogonia of non-obstructive azoospermia (NOA) patients when compared with obstructive azoospermia (OA) patients [64]. In our study, exclusive variants were discovered in both mature and pre-miRNA sequences of hsa-mir-296 and hsa-miR-296-3p in asthenozoospermic and oligozoospermic samples. Another miRNA of interest is hsa-mir-518d, an exclusive variant identified in asthenozoospermic samples. hsa-miR-518d-5p is differentially expressed in spermatozoa samples from normozoospermic fertile versus infertile men [63], and its role in male infertility is further underscored by differential expression in testicular samples of Sertoli cell-only syndrome (SCOS) patients compared to OA patients [65]. Furthermore, hsa-miR-200b-3p has also been found to be abundant in sperm samples [66], and an exclusive variant was identified in the pre-miRNA region of hsa-miR-200b in teratozoospermic samples in the present study. Similarly, exclusive variants were found in both mature and pre-miRNA regions of hsa-mir-449b and hsa-miR-449b-5p in samples from asthenozoospermia and oligozoospermia. This miRNA has been linked to male infertility, showing differential expression in testicular biopsies of infertile patients with impaired spermatogenesis [42,67]. Finally, numerous variants were discovered in oligozoospermic samples within hsa-mir-548ad, hsa-mir-548a-1, hsa-mir-548h-4, and hsa-miR-548h-3p. Studies show that hsa-mir-548 family members are variably expressed in the reproductive tract and likely fulfill different regulatory roles [68]. Therefore, these findings not only confirm prior studies on the role of specific miRNAs in male infertility but also pave the way for future diagnostic and therapeutic interventions after further validation.

### 4.2. Molecular Mechanisms and Pathways Affected by miRNA Variants

The exclusive variants identified in miRNA and pre-miRNA regions have the potential to alter miRNA functionality and, consequently, gene regulation. To understand the impact of these variants on male infertility, we investigated the genes targeted by affected miRNAs, focusing on pathways and molecular mechanisms that could be disrupted.

At first, the regulation of transcription by RNA polymerase II emerged as a critical biological process affected in teratozoospermia, asthenozoospermia, and oligozoospermia. Studies highlight RNA polymerase II’s involvement in the transcriptional regulation of meiosis and sperm differentiation, underscoring its role in maintaining normal reproductive functions [69,70]. Consequently, abnormalities in transcription regulation could lead to altered gene expression essential for sperm development and function, potentially explaining the phenotypic variations observed in these conditions.

Furthermore, the recurrent identification of cancer-related pathways in our study provides compelling evidence of shared molecular pathways that may inadvertently influence reproductive functions. This observation suggests that oncogenic pathways, when dysregulated, could have secondary effects on reproductive health, possibly through mechanisms controlling cell growth and survival. It should be noted that this is not the first study that suggests a link between male infertility and cancer. Kyrgiafini et al. (2022) [71] identified several long noncoding RNAs (lncRNAs) that are deregulated in male infertility and also play a role in cancer. Recent research has also increasingly focused on the critical role of miRNAs in both male infertility and reproductive cancers, highlighting a complex genetic and molecular interplay. In the context of male infertility, miRNAs influence key reproductive functions, including spermatogenesis, sperm motility, and overall reproductive health. Simultaneously, the dysregulation of these miRNAs is also associated with the development of cancers, suggesting that similar molecular pathways may be disrupted in both conditions [14,72]. Therefore, although the link between male infertility and cancer is well established, future studies are required to fully elucidate the mechanisms behind this link. Understanding these connections could pave the way for cross-disciplinary approaches that target these pathways to treat both cancer and infertility.

Additionally, several signaling pathways, particularly the TGF-β and ErbB pathways, were found to be deregulated by affected miRNAs. More specifically, the TGF-β and ErbB signaling pathways are implicated in various cellular functions, including cell proliferation, differentiation, and apoptosis, all of which are essential for normal spermatogenesis. The specific pathways have also been implicated in playing an important role in Sertoli cells [73]. Disruption in these signaling pathways, as indicated by our findings, suggests a mechanistic pathway that may lead to impaired spermatogenic function. Therefore, given their roles, alterations in these pathways could disrupt the cellular environment of the testes, leading to infertility.

Significantly, our findings also involve the p53 signaling pathway, known for regulating the cell cycle and inducing apoptosis in response to cellular stress and DNA damage [74]. More specifically, the p53 protein, highly expressed in testis, spermatogonia [75], and primary spermatocytes, plays a pivotal role in ensuring the quality and quantity of mature spermatozoa [76]. Thus, aberrations in this pathway could lead to defective spermatogenic processes and increased apoptosis within the testes, contributing to infertility.

Thus, the above findings provide insights into the mechanisms involved in male infertility and highlight potential therapeutic targets through the regulation of these pathways.

### 4.3. Exclusive Variants in miRNA Regions

The primary aim of this study was to identify miRNA variants in miRNAs found exclusively in infertile men with the potential to contribute to the observed phenotypes. In this study, we reported such variants across all categories of infertility studied, including teratozoospermia, asthenozoospermia, and oligozoospermia. In general, variants in pre-miRNAs or mature miRNAs can significantly impact gene expression by altering miRNA maturation and function, thus disrupting their regulatory roles. More specifically, variants in pre-miRNAs can affect the processing of these molecules into mature miRNAs, potentially changing the miRNA’s stability, its incorporation into the RNA-induced silencing complex (RISC), or its specificity for target mRNAs. Consequently, a variant may lead to decreased miRNA levels, reducing its regulatory function, or it could result in the production of a miRNA with altered target specificity, potentially silencing genes not regulated by the wild-type miRNA [17,18]. Similarly, variants in mature miRNAs can directly affect their binding to target mRNAs. Such changes can either enhance or reduce the affinity of miRNA–mRNA interactions, leading to aberrant gene expression [17,18,77].

Although none of the SNPs identified has previously been associated with male infertility, some of them have been investigated for their impacts on other pathological conditions. More specifically, rs17797090, common across all infertile groups (teratozoospermia, asthenozoospermia, oligozoospermia), may decrease hsa-mir-3652 production, according to the study of Gong et al. (2011) [78]. Furthermore, rs2682818, found in teratozoospermic and asthenozoospermic men, has been studied for its functional impact, where the variant T allele was shown to reduce levels of mature miR-618, potentially leading to the deregulation of miR-618-controlled pathways [79]. In general, this variant in the MIR618 gene has been associated with the regulation of miR-618 expression and has implications for susceptibility and progression of various diseases, including breast cancer, colorectal carcinoma, and metastatic colon cancer [80,81,82,83]. Additionally, variants such as rs72563729 [84], and rs12803915 [85] have been associated with various types of cancer. Notably, another SNP, rs10061133, found in asthenozoospermic and teratozoospermic men, has been associated with premature ovarian insufficiency [86], idiopathic recurrent pregnancy loss [87], and recurrent implantation failure [88], suggesting a potential role in male fertility, too.

Therefore, all the variants reported in this study are significant and warrant further investigation in future studies on male infertility. Specifically, the six variants (rs17797090, rs1844035, rs7210937, rs451887, rs12233076, rs6787734) that were common across the infertile groups could even have the potential to be used as biomarkers. Finally, the variants identified in the present study with functional significance could also be investigated for their role in male infertility.

### 4.4. Study Limitations and Strengths

Regarding the limitations of our study, it is important to note that it primarily relied on bioinformatics approaches and in silico analyses to assess the role of miRNA variants in male infertility. Furthermore, the research was conducted on a relatively small cohort consisting of five individuals each for teratozoospermia, asthenozoospermia, and oligozoospermia, and ten for normozoospermia. The small sample size may limit the generalizability of our findings. Regarding our sample, it should also be noted that all participants in this study were volunteers, contributing to a diverse sample representing various age groups and lifestyle habits. While this diversity enriches the dataset, it may also introduce variability that impacts the findings. Although significant age differences were observed between some groups, notably between asthenozoospermic and oligozoospermic, no significant difference was found between the normozoospermic group and any other infertile group. The primary objective of our study was to compare the normozoospermic group with the infertile groups (asthenozoospermic, oligozoospermic, and teratozoospermic) to identify unique variants. Thus, as the age difference between normozoospermic men and any infertile group is not statistically significant, this ensures that our main comparisons remain valid. However, it should be noted that men over forty years old were included in this study. Research indicates that age is associated with abnormal semen parameters [89]. While similar age variability was observed across the study groups, this factor should be considered when addressing the limitations of the present study. Furthermore, the analysis indicated no statistically significant differences in smoking or alcohol consumption habits between the groups, despite apparent variations in percentages. The lack of significance may be attributed to the small sample sizes within each subgroup. Therefore, all of the above should be considered when examining the limitations of this study.

However, to mitigate these limitations, we employed whole genome sequencing, which, due to its comprehensive nature, allows for the examination of the entire genetic landscape, providing a detailed overview of potential genetic contributors to male infertility. Notably, studies utilizing WGS to investigate male infertility are scarce, making our research particularly significant in this field. Additionally, we enhanced the robustness of our findings by utilizing a broad spectrum of databases and varied analytical tools, including SNPnexus [27], 3DSNP 2.0 [33], and RegulomeDB databases [34], among others. We also adhered to stringent selection criteria, particularly regarding scores from RegulomeDB [34] and 3DSNP 2.0 [33], to enhance the reliability of our results. Furthermore, to ensure the accuracy and consistency of our conclusions, we based our analyses on experimentally validated interactions to identify connections between mRNAs and miRNAs, according to miRTargetLink 2.0 [28]. We also validated our findings after annotation by focusing solely on variants mapped to miRNAs according to miRNASNP v3 [26]. Finally, this study is among the first to explore variants in miRNA genes and their association with male infertility, highlighting the need for such research as a starting point for more extensive future investigations in the field of male infertility.

### 4.5. Future Studies

The findings from our current research pave the way for further studies in understanding male infertility. Future research should focus on extending the analysis of miRNA variants, identified exclusively in infertile men, to larger cohorts. This would help validate the impact of these variants on infertility phenotypes. Conducting genome-wide association studies (GWAS) could also substantiate the significance of these identified variants and their correlation with male infertility and its specific subtypes. Additionally, experimental verification of the functional roles of these miRNAs should be pursued through in vitro and in vivo studies to establish a direct causal relationship between specific miRNA dysregulation and infertility.

Moreover, integrating the study of miRNA variants with broader genomic, transcriptomic, and proteomic analyses could provide deeper insights into the complex interactions and regulatory networks that affect spermatogenesis and male infertility.

Last but not least, given the established link between miRNA dysregulation in infertility and cancer, interdisciplinary studies that bridge oncology and reproductive biology could lead to significant breakthroughs. Such research could not only enhance our understanding of these conditions but also lead to the development of novel therapeutic approaches that address the underlying genetic and molecular mechanisms.

## 5. Conclusions

This study is among the first to specifically investigate miRNA variants in male infertility, particularly focusing on its distinct subtypes: teratozoospermia, asthenozoospermia, and oligozoospermia.

In this study, our primary aim was to provide a comprehensive roadmap for future investigations by identifying unique genetic variants in miRNAs. Recently, many miRNAs have been reported to be differentially expressed (DE) in male infertility [90,91]; thus, the approach used here allows us to prioritize miRNAs for further functional studies. Furthermore, male infertility remains an understudied area, with only a limited number of variants currently linked to this condition. Our study addresses this gap by identifying novel variants, found only in infertile men, that have the potential to contribute to the phenotype and serve as biomarkers. Additionally, as studies associate different assisted reproductive technology (ART) outcomes with genetic background [92,93], it is crucial to report as many variants associated with male infertility as possible to enhance future studies. From this perspective, whole-genome sequencing (WGS) can provide a comprehensive view, capturing many variants that are often missed by genome-wide association studies (GWAS) and SNP chips [94].

In summary, our preliminary study provides a valuable roadmap for future research by identifying and prioritizing miRNAs with genetic variants found only in infertile men. However, keeping in mind the limitations of our study, we strongly encourage functional studies to validate the roles of these variants and miRNAs. This effort will enhance our understanding of male infertility and pave the way for the development of novel diagnostic tools and therapeutic strategies.

## Figures and Tables

**Table 1:** Demographic details of study participants.

Demographics	Normozoospermic (*n* = 10)	Teratozoospermic (*n* = 5)	Asthenozoospermic (*n* = 5)	Oligozoospermic (*n* = 5)	*p*-Value
Age	28–53 Mean = 36.4 (SD = 7.2)	31–49 Mean = 38 (SD = 6.82)	21–32 Mean = 29 (SD = 5.07)	34–41 Mean = 39 (SD = 2.83)	0.049717 (ANOVA) 0.037 (Tukey’s test, Asthenoz.–Oligoz.)
Body Mass Index (BMI)	19.5–40.4 Mean = 26.97 (SD = 6.07)	24.8–33 Mean = 29.24 (SD = 3.31)	20.5–32.3 Mean = 25.33 (SD = 5.28)	26.5–36.3 Mean = 31.1 (SD = 4.43)	0.362778 (ANOVA)
Smoking	30% Not Smoking, 70% Smoking	60% Not Smoking, 40% Smoking	60% Not Smoking, 40% Smoking	40% Not Smoking, 60% Smoking	0.4148 (chi-square test)
Alcohol	100% ≤ 2 drinks/week	80% ≤ 2 drinks/week	80% ≤ 2 drinks/week	80% ≤ 2 drinks/week	0.560632 (chi-square test)

**Table 2:** Variants in pre-miRNAs, mature miRNAs, and seed regions according to miRNASNP v3 [26] and SNPnexus [27] for teratozoospermia.

Variant	Gene	miRNAs	Region
rs17797090	MIR3652	hsa-mir-3652	pre-miRNA
rs2682818	MIR618	hsa-mir-618	pre-miRNA
rs35170395	MIR3171	hsa-mir-3171	pre-miRNA
rs7210937	MIR1269B	hsa-mir-1269b	pre-miRNA and seed region
rs10670323	MIR516B2	hsa-mir-516b-2	pre-miRNA
rs72563729	MIR200B	hsa-mir-200b	pre-miRNA
rs74904371	MIR2682	hsa-mir-2682, hsa-miR-2682-3p	pre-miRNA and seed region
rs451887	MIR5692B	hsa-mir-5692b	pre-miRNA and seed region
rs5996397	MIR650	hsa-mir-650	pre-miRNA
rs200194626	MIR663B	hsa-mir-663b	pre-miRNA
rs199671138	MIR663B	hsa-mir-663b	pre-miRNA
rs12233076	MIR4441	hsa-mir-4441	pre-miRNA
rs78831152	MIR4789	hsa-mir-4789	pre-miRNA
rs6787734	MIR3135A	hsa-mir-3135a	pre-miRNA
rs142342924	MIR3135A	hsa-mir-3135a	pre-miRNA
rs4994089	MIR548U	hsa-mir-548u	pre-miRNA
rs374409015	MIR4467	hsa-mir-4467	pre-miRNA and seed region
rs6943868	MIR3683	hsa-mir-3683	pre-miRNA
rs1844035	MIR4477B	hsa-mir-4477a, hsa-mir-4477b	pre-miRNA

**Table 3:** Variants in pre-miRNAs, mature miRNAs, and seed regions according to miRNASNP v3 [26] and SNPnexus [27] for asthenozoospermia.

Variants	Genes	miRNAs	Region
rs17091403	MIR2110	hsa-mir-2110	pre-miRNA
rs12803915	MIR612	hsa-mir-612	pre-miRNA
rs17797090	MIR3652	hsa-mir-3652	pre-miRNA
rs2682818	MIR618	hsa-mir-618	pre-miRNA
rs11435035	MIR5094	hsa-mir-5094	pre-miRNA
rs7210937	MIR1269B	hsa-miR-1269b	pre-miRNA and seed region
rs74704964	MIR518D	hsa-mir-518d	pre-miRNA
rs117258475	MIR296	hsa-mir-296, hsa-miR-296-3p	mature miRNA and pre-miRNA
rs451887	MIR5692B	hsa-mir-5692b	pre-miRNA and seed region
rs5996397	MIR650	hsa-mir-650	pre-miRNA
rs12233076	MIR4441	hsa-mir-4441	pre-miRNA
rs78832554	MIR4786	hsa-mir-4786	pre-miRNA
rs6787734	MIR3135A	hsa-mir-3135a	pre-miRNA
rs142342924	MIR3135A	hsa-mir-3135a	pre-miRNA
rs10575780	MIR3938	hsa-mir-3938	pre-miRNA
rs10061133	MIR449B	hsa-mir-449b, hsa-miR-449b-5p	mature miRNA and pre-miRNA
rs73024232	MIR3939	hsa-mir-3939	pre-miRNA and seed region
rs67030829	MIR4645	hsa-mir-4645	pre-miRNA
rs4994089	MIR548U	hsa-mir-548u	pre-miRNA
rs921372085	MIR4656	hsa-mir-4656	pre-miRNA
rs12549434	MIR5680	hsa-mir-5680	pre-miRNA
rs113454901	MIR3689D1	hsa-mir-3689d-1	pre-miRNA
rs1844035	MIR4477B	hsa-mir-4477a, hsa-mir-4477b	pre-miRNA
rs356125	MIR2278	hsa-mir-2278	pre-miRNA

**Table 4:** Variants in pre-miRNAs, mature miRNAs, and seed regions according to miRNASNP v3 [26] and SNPnexus [27] for oligozoospermia.

Variants	Genes	miRNAs	Region
rs17091403	MIR2110	hsa-mir-2110	pre-miRNA
rs12803915	MIR612	hsa-mir-612	pre-miRNA
rs17797090	MIR3652	hsa-mir-3652	pre-miRNA
rs191393746	MIR1538	hsa-mir-1538	pre-miRNA
rs7210937	MIR1269B	hsa-miR-1269b	pre-miRNA and seed region
rs72855836	MIR3976	hsa-mir-3976	pre-miRNA
rs56013413	MIR520H	hsa-mir-520h	pre-miRNA
rs117258475	MIR296	hsa-mir-296, hsa-miR-296-3p	mature miRNA and pre-miRNA
rs451887	MIR5692B	hsa-mir-5692b	pre-miRNA and seed region
rs200194626	MIR663B	hsa-mir-663b	pre-miRNA
rs199671138	MIR663B	hsa-mir-663b	pre-miRNA
rs767805489	MIR1302-4	hsa-mir-1302-4	pre-miRNA
rs12233076	MIR4441	hsa-mir-4441	pre-miRNA
rs918690276	MIR548AD	hsa-mir-548ad	pre-miRNA
rs114803590	MIR559	hsa-mir-559	pre-miRNA
rs78831152	MIR4789	hsa-mir-4789	pre-miRNA
rs6787734	MIR3135A	hsa-mir-3135a	pre-miRNA
rs772572114	MIR3142	hsa-miR-3142	mature miRNA and pre-miRNA
rs10061133	MIR449B	hsa-mir-449b, hsa-miR-449b-5p	mature miRNA and pre-miRNA
rs73024232	MIR3939	hsa-miR-3939	pre-miRNA and seed region
rs12197631	MIR548A1	hsa-mir-548a-1	pre-miRNA
rs6943868	MIR3683	hsa-mir-3683	pre-miRNA
rs12549434	MIR5680	hsa-mir-5680	pre-miRNA
rs73235381	MIR548H4	hsa-mir-548h-4, hsa-miR-548h-3p	mature miRNA and pre-miRNA
rs184537764	MIR548H4	hsa-mir-548h-4	pre-miRNA
rs1844035	MIR4477B	hsa-mir-4477a, hsa-mir-4477b	pre-miRNA
rs356125	MIR2278	hsa-mir-2278	pre-miRNA

**Table 5:** Variants mapped in miRNAs with regulatory potential according to the 3DSNP 2.0 [33] and RegulomeDB databases [34] for teratozoospermia; MAF: Minor Allele Frequency. Significance is indicated in bold for both RegulomeDB Rank and 3DSNP score.

Variants	MAF	miRNAs	Region	RegulomeDB Rank	3DSNP Score
rs17797090	0.17	hsa-mir-3652	pre-miRNA	**1f**	**203.36**
rs2682818	0.42	hsa-mir-618	pre-miRNA	4	**18.24**
rs10670323	0.37	hsa-mir-516b-2	pre-miRNA	7	**17.23**
rs72563729	0.04	hsa-mir-200b	pre-miRNA	4	**13.67**
rs74904371	0.08	hsa-mir-2682, hsa-miR-2682-3p	pre-miRNA and seed region	4	**65.09**
rs5996397	0.38	hsa-mir-650	pre-miRNA	**1d**	**11.19**
rs6787734	0.50	hsa-mir-3135a	pre-miRNA	**1d**	3.09
rs374409015	0.08	hsa-mir-4467	pre-miRNA and seed region	**1f**	3.82

**Table 6:** Variants mapped in miRNAs with regulatory potential according to the 3DSNP 2.0 [33] and RegulomeDB databases [34] for asthenozoospermia; MAF: Minor Allele Frequency. Significance is indicated in bold for both RegulomeDB Rank and 3DSNP score.

Variants	MAF	miRNAs	Region	RegulomeDB Rank	3DSNP Score
rs17091403	0.12	hsa-mir-2110	pre-miRNA	**1f**	**205.72**
rs12803915	0.33	hsa-mir-612	pre-miRNA	**1f**	**56.74**
rs17797090	0.17	hsa-mir-3652	pre-miRNA	**1f**	**203.36**
rs2682818	0.42	hsa-mir-618	pre-miRNA	4	**18.24**
rs11435035	0.48	hsa-mir-5094	pre-miRNA	**2b**	7.04
rs74704964	0.07	hsa-mir-518d	pre-miRNA	5	**17.68**
rs5996397	0.38	hsa-mir-650	pre-miRNA	**1d**	**11.19**
rs6787734	0.50	hsa-mir-3135a	pre-miRNA	**1d**	3.09
rs10061133	0.34	hsa-mir-449b, hsa-miR-449b-5p	mature miRNA and pre-miRNA	**1f**	5.52
rs73024232	0.38	hsa-mir-3939	pre-miRNA and seed region	4	**201.97**
rs67030829	0.03	hsa-mir-4645	pre-miRNA	4	**213.74**
rs356125	0.13	hsa-mir-2278	pre-miRNA	**1f**	4.85

**Table 7:** Variants mapped in miRNAs with regulatory potential according to the 3DSNP 2.0 [33] and RegulomeDB databases [34] for oligozoospermia; MAF: Minor Allele Frequency. Significance is indicated in bold for both RegulomeDB Rank and 3DSNP score.

Variants	MAF	miRNAs	Region	RegulomeDB Rank	3DSNP Score
rs17091403	0.12	hsa-mir-2110	pre-miRNA	**1f**	**205.72**
rs12803915	0.33	hsa-mir-612	pre-miRNA	**1f**	**56.74**
rs17797090	0.17	hsa-mir-3652	pre-miRNA	**1f**	**203.36**
rs191393746	0.04	hsa-mir-1538	pre-miRNA	4	**204.49**
rs56013413	0.30	hsa-mir-520h	pre-miRNA	7	**20.62**
rs767805489	<0.01	hsa-mir-1302-4	pre-miRNA	**2b**	1.49
rs114803590	0.05	hsa-mir-559	pre-miRNA	**1f**	2.20
rs6787734	0.50	hsa-mir-3135a	pre-miRNA	**1d**	3.09
rs772572114	<0.01	hsa-mir-3142	mature miRNA and pre-miRNA	**1f**	6.22
rs10061133	0.34	hsa-mir-449b, hsa-miR-449b-5p	mature miRNA and pre-miRNA	**1f**	5.52
rs73024232	0.38	hsa-miR-3939	pre-miRNA and seed region	4	**201.97**
rs184537764	0.11	hsa-mir-548h-4	pre-miRNA	**1f**	2.30
rs356125	0.13	hsa-mir-2278	pre-miRNA	**1f**	4.85

**Table 8:** Common miRNA variants across different categories of infertile men; MAF: Minor Allele Frequency; Terato: Teratozoospermia; Astheno: Asthenozoospermia; Oligo: Oligozoospermia.

Variants	MAF	miRNAs	Region	Infertility Category
rs17797090	0.17	hsa-mir-3652	pre-miRNA	Terato–Astheno–Oligo
rs1844035	0.50	hsa-mir-4477a, hsa-mir-4477b	pre-miRNA	Terato–Astheno–Oligo
rs7210937	0.50	hsa-mir-1269b	pre-miRNA and seed region	Terato–Astheno–Oligo
rs451887	0.50	hsa-mir-5692b	pre-miRNA and seed region	Terato–Astheno–Oligo
rs12233076	0.49	hsa-mir-4441	pre-miRNA	Terato–Astheno–Oligo
rs6787734	0.50	hsa-mir-3135a	pre-miRNA	Terato–Astheno–Oligo
rs5996397	0.38	hsa-mir-650	pre-miRNA	Terato–Astheno
rs142342924	0.27	hsa-mir-3135a	pre-miRNA	Terato–Astheno
rs4994089	0.25	hsa-mir-548u	pre-miRNA	Terato–Astheno
rs2682818	0.42	hsa-mir-618	pre-miRNA	Terato–Astheno
rs200194626	<0.01	hsa-mir-663b	pre-miRNA	Terato–Oligo
rs199671138	<0.01	hsa-mir-663b	pre-miRNA	Terato–Oligo
rs78831152	0.26	hsa-mir-4789	pre-miRNA	Terato–Oligo
rs6943868	0.48	hsa-mir-3683	pre-miRNA	Terato–Oligo
rs17091403	0.12	hsa-mir-2110	pre-miRNA	Astheno–Oligo
rs12803915	0.33	hsa-mir-612	pre-miRNA	Astheno–Oligo
rs117258475	0.02	hsa-mir-296, hsa-miR-296-3p	mature miRNA and pre-miRNA	Astheno–Oligo
rs10061133	0.34	hsa-mir-449b, hsa-miR-449b-5p	mature miRNA and pre-miRNA	Astheno–Oligo
rs73024232	0.38	hsa-mir-3939	pre-miRNA and seed region	Astheno–Oligo
rs12549434	0.19	hsa-mir-5680	pre-miRNA	Astheno–Oligo
rs356125	0.13	hsa-mir-2278	pre-miRNA	Astheno–Oligo

**Table 9:** Validation of common variants detected by WGS in an independent cohort. Genotyping results, with *p*-values indicating the frequency difference between infertile and normozoospermic men.

Variants	*p*-Value
rs1844035	0.023
rs12233076	0.004
rs142342924	0.002
rs6943868	0.02
rs117258475	0.041
rs73024232	0.015
rs12549434	0.006
rs356125	0.036

**Table 10:** miRNAs in which common variants were identified among infertile men and differential expression based on a literature search.

miRNAs	Tissue	Comparison	Function	References
hsa-mir-296	Semen	Fertile patients vs. teratozoospermic patients	NA	Corral-Vazquez et al. (2019) [38]
hsa-mir-296	Semen	Smokers vs. non-smokers	Downregulated	Metzler-Guillemain et al. (2015) [39]
hsa-mir-449b	Semen	Infertile vs. fertile men	Downregulated	Najafipour et al. (2021) [40]
hsa-mir-449b	Semen	Men of couples undergoing ART	Positively associated with sperm DNA fragmentation	Confitti et al. (2023) [41]
hsa-mir-449b	Testicular tissue	Patients with germ cell arrest vs. normal	Downregulated	Abu-Halima et al. (2014) [42]
hsa-mir-548u	Seminal plasma	Patients with Sertoli cell-only syndrome (SCOS) vs. normal fertile controls	Upregulated	Zhang et al. (2021) [43]
hsa-mir-618	Semen	Comparison between IVF patients’ groups with different fertilization, effective embryo rate and high-quality embryo rate	NA	Xu et al. (2020) [44]
hsa-mir-663b	Seminal microvesicles	Patients before vasectomy and after vasectomy	Upregulated	Belleannée et al. 2013 [45]
hsa-mir-663b	Semen	Ten individuals with normal seminogram, standard karyotype, and proven fertility	Most stable miRNA (top 10)	Salas-Huetos et al. (2014) [46]
hsa-mir-650	Testicular tissue	Seminoma tissues vs. normal tissues	Downregulated	Wang et al. (2019) [47]

## Data Availability

Whole-genome sequencing data of normozoospermic and teratozoospermic men used in this study are available through SRA (BioProject ID PRJNA875412, http://www.ncbi.nlm.nih.gov/bioproject/875412, accessed on 7 September 2024).

## Supplementary Materials

The following supporting information can be downloaded at: https://www.mdpi.com/article/10.3390/genes15111393/s1, Table S1: Variants identified in miRNA genes after annotation using VEP for teratozoospermia, asthenozoospermia and oligozoospermia; Table S2: Experimental miRNA–mRNA interactions for teratozoospermia, asthenozoospermia and oligozoospermia according to miRTargetLink 2.0; Table S3: 3DSNP scores for identified variants in miRNA genes; Table S4: RegulomeDB Ranks for identified variants in miRNA genes; Figure S1: Significant GO Cellular Components terms of target genes of affected miRNAs in teratozoospermia; Figure S2: Significant GO Molecular Function terms of target genes of affected miRNAs in teratozoospermia; Figure S3: Significant GO Cellular Components terms of target genes of affected miRNAs in asthenozoospermia; Figure S4: Significant GO Molecular Function terms of target genes of affected miRNAs in asthenozoospermia; Figure S5: Significant GO Cellular Components terms of target genes of affected miRNAs in oligozoospermia; Figure S6: Significant GO Molecular Function terms of target genes of affected miRNAs in oligozoospermia.

## References

[1] Agarwal A., Mulgund A., Hamada A., Chyatte M.R. (2015). A Unique View on Male Infertility around the Globe. Reprod. Biol. Endocrinol..

[2] Agarwal A., Baskaran S., Parekh N., Cho C.L., Henkel R., Vij S., Arafa M., Panner Selvam M.K., Shah R. (2021). Male Infertility. Lancet.

[3] Krausz C., Riera-Escamilla A. (2018). Genetics of Male Infertility. Nat. Rev. Urol..

[4] Huang B., Wang Z., Kong Y., Jin M., Ma L. (2023). Global, Regional and National Burden of Male Infertility in 204 Countries and Territories Between 1990 and 2019: An Analysis of Global Burden of Disease Study. BMC Public Health.

[5] Skinner M.K. (2018). Encyclopedia of Reproduction.

[6] Esteller M. (2011). Non-Coding RNAs in Human Disease. Nat. Rev. Genet..

[7] Condrat C.E., Thompson D.C., Barbu M.G., Bugnar O.L., Boboc A., Cretoiu D., Suciu N., Cretoiu S.M., Voinea S.C. (2020). MiRNAs as Biomarkers in Disease: Latest Findings Regarding Their Role in Diagnosis and Prognosis. Cells.

[8] Ardekani A.M., Naeini M.M. (2010). The Role of MicroRNAs in Human Diseases. Avicenna J. Med. Biotechnol..

[9] O’Brien J., Hayder H., Zayed Y., Peng C. (2018). Overview of MicroRNA Biogenesis, Mechanisms of Actions, and Circulation. Front. Endocrinol..

[10] Plotnikova O., Baranova A., Skoblov M. (2019). Comprehensive Analysis of Human MicroRNA–MRNA Interactome. Front. Genet..

[11] Wu S., Huang S., Ding J., Zhao Y., Liang L., Liu T., Zhan R., He X. (2010). Multiple MicroRNAs Modulate P21Cip1/Waf1 Expression by Directly Targeting Its 3’ Untranslated Region. Oncogene.

[12] Xu P., Wu Q., Yu J., Rao Y., Kou Z., Fang G., Shi X., Liu W., Han H. (2020). A Systematic Way to Infer the Regulation Relations of MiRNAs on Target Genes and Critical MiRNAs in Cancers. Front. Genet..

[13] Liu B., Li J., Cairns M.J. (2014). Identifying MiRNAs, Targets and Functions. Brief. Bioinform..

[14] Khawar M.B., Mehmood R., Roohi N. (2019). MicroRNAs: Recent Insights towards Their Role in Male Infertility and Reproductive Cancers. Bosn. J. Basic Med. Sci..

[15] Mukherjee A., Koli S., Reddy K.V.R. (2014). Regulatory Non-Coding Transcripts in Spermatogenesis: Shedding Light on ‘Dark Matter’. Andrology.

[16] Salas-Huetos A., James E.R., Aston K.I., Carrell D.T., Jenkins T.G., Yeste M. (2020). The Role of MiRNAs in Male Human Reproduction: A Systematic Review. Andrology.

[17] Machowska M., Galka-Marciniak P., Kozlowski P. (2022). Consequences of Genetic Variants in MiRNA Genes. Comput. Struct. Biotechnol. J..

[18] Moszyńska A., Gebert M., Collawn J.F., Bartoszewski R. (2017). SNPs in MicroRNA Target Sites and Their Potential Role in Human Disease. Open Biol..

[19] Andrew S. FastQC: A Quality Control Tool for High Throughput Sequence Data [Online] 2010. https://www.bioinformatics.babraham.ac.uk/projects/fastqc/.

[20] Bolger A.M., Lohse M., Usadel B. (2014). Trimmomatic: A Flexible Trimmer for Illumina Sequence Data. Bioinformatics.

[21] Martin F.J., Amode M.R., Aneja A., Austine-Orimoloye O., Azov A.G., Barnes I., Becker A., Bennett R., Berry A., Bhai J. (2023). Ensembl 2023. Nucleic Acids Res..

[22] Li H., Durbin R. (2009). Fast and Accurate Short Read Alignment with Burrows-Wheeler Transform. Bioinformatics.

[23] Danecek P., Bonfield J.K., Liddle J., Marshall J., Ohan V., Pollard M.O., Whitwham A., Keane T., McCarthy S.A., Davies R.M. (2021). Twelve Years of SAMtools and BCFtools. Gigascience.

[24] Garrison E., Marth G. (2012). Haplotype-Based Variant Detection from Short-Read Sequencing. arXiv.

[25] McLaren W., Gil L., Hunt S.E., Riat H.S., Ritchie G.R.S., Thormann A., Flicek P., Cunningham F. (2016). The Ensembl Variant Effect Predictor. Genome Biol..

[26] Liu C.J., Fu X., Xia M., Zhang Q., Gu Z., Guo A.Y. (2021). MiRNASNP-v3: A Comprehensive Database for SNPs and Disease-Related Variations in MiRNAs and MiRNA Targets. Nucleic Acids Res..

[27] Oscanoa J., Sivapalan L., Gadaleta E., Dayem Ullah A.Z., Lemoine N.R., Chelala C. (2020). SNPnexus: A Web Server for Functional Annotation of Human Genome Sequence Variation (2020 Update). Nucleic Acids Res..

[28] Kern F., Aparicio-Puerta E., Li Y., Fehlmann T., Kehl T., Wagner V., Ray K., Ludwig N., Lenhof H.P., Meese E. (2021). MiRTargetLink 2.0—Interactive MiRNA Target Gene and Target Pathway Networks. Nucleic Acids Res..

[29] Ashburner M., Ball C.A., Blake J.A., Botstein D., Butler H., Cherry J.M., Davis A.P., Dolinski K., Dwight S.S., Eppig J.T. (2000). Gene Ontology: Tool for the Unification of Biology. Nat. Genet..

[30] Consortium T.G.O., Aleksander S.A., Balhoff J., Carbon S., Cherry J.M., Drabkin H.J., Ebert D., Feuermann M., Gaudet P., Harris N.L. (2023). The Gene Ontology Knowledgebase in 2023. Genetics.

[31] Kanehisa M., Furumichi M., Sato Y., Kawashima M., Ishiguro-Watanabe M. (2023). KEGG for Taxonomy-Based Analysis of Pathways and Genomes. Nucleic Acids Res..

[32] Ge S.X., Jung D., Jung D., Yao R. (2020). ShinyGO: A Graphical Gene-Set Enrichment Tool for Animals and Plants. Bioinformatics.

[33] Quan C., Ping J., Lu H., Zhou G., Lu Y. (2022). 3DSNP 2.0: Update and Expansion of the Noncoding Genomic Variant Annotation Database. Nucleic Acids Res..

[34] Boyle A.P., Hong E.L., Hariharan M., Cheng Y., Schaub M.A., Kasowski M., Karczewski K.J., Park J., Hitz B.C., Weng S. (2012). Annotation of Functional Variation in Personal Genomes Using RegulomeDB. Genome Res..

[35] Chatziparasidou A., Kyrgiafini M.A., Sarafidou T., Moutou K.A., Mamuris Z. (2024). Genetic Insights into Azoospermia and Severe Oligozoospermia: Discovering Seven SNPs Through GWAS and In Silico Analysis. Curr. Issues Mol. Biol..

[36] Purcell S., Neale B., Todd-Brown K., Thomas L., Ferreira M.A.R., Bender D., Maller J., Sklar P., De Bakker P.I.W., Daly M.J. (2007). PLINK: A Tool Set for Whole-Genome Association and Population-Based Linkage Analyses. Am. J. Hum. Genet..

[37] Lewis B.P., Shih I.H., Jones-Rhoades M.W., Bartel D.P., Burge C.B. (2003). Prediction of Mammalian MicroRNA Targets. Cell.

[38] Corral-Vazquez C., Salas-Huetos A., Blanco J., Vidal F., Sarrate Z., Anton E. (2019). Sperm MicroRNA Pairs: New Perspectives in the Search for Male Fertility Biomarkers. Fertil. Steril..

[39] Metzler-Guillemain C., Victorero G., Lepoivre C., Bergon A., Yammine M., Perrin J., Sari-Minodier I., Boulanger N., Rihet P., Nguyen C. (2015). Sperm MRNAs and MicroRNAs as Candidate Markers for the Impact of Toxicants on Human Spermatogenesis: An Application to Tobacco Smoking. Syst. Biol. Reprod. Med..

[40] Najafipour R., Momeni A., Yousefipour F., Mousavi S., Moghbelinejad S. (2021). Underexpression of Hsa-MiR-449 Family and Their Promoter Hypermethylation in Infertile Men: A Case-Control Study. Int. J. Reprod. Biomed..

[41] Conflitti A.C., Cicolani G., Buonacquisto A., Pallotti F., Faja F., Bianchini S., Blaconà G., Bruno S.M., Linari A., Lucarelli M. (2023). Sperm DNA Fragmentation and Sperm-Borne MiRNAs: Molecular Biomarkers of Embryo Development?. Int. J. Mol. Sci..

[42] Abu-Halima M., Backes C., Leidinger P., Keller A., Lubbad A.M., Hammadeh M., Meese E. (2014). MicroRNA Expression Profiles in Human Testicular Tissues of Infertile Men with Different Histopathologic Patterns. Fertil. Steril..

[43] Zhang W., Zhang Y., Zhao M., Ding N., Yan L., Chen J., Gao L., Zhang G., Sun X., Gu Y. (2021). MicroRNA Expression Profiles in the Seminal Plasma of Nonobstructive Azoospermia Patients with Different Histopathologic Patterns. Fertil. Steril..

[44] Xu H., Wang X., Wang Z., Li J., Xu Z., Miao M., Chen G., Lei X., Wu J., Shi H. (2020). MicroRNA Expression Profile Analysis in Sperm Reveals Hsa-Mir-191 as an Auspicious Omen of In Vitro Fertilization. BMC Genom..

[45] Belleannée C., Légaré C., Calvo É., Thimon V., Sullivan R. (2013). MicroRNA Signature Is Altered in Both Human Epididymis and Seminal Microvesicles Following Vasectomy. Hum. Reprod..

[46] Salas-Huetos A., Blanco J., Vidal F., Mercader J.M., Garrido N., Anton E. (2014). New Insights into the Expression Profile and Function of Micro-Ribonucleic Acid in Human Spermatozoa. Fertil. Steril..

[47] Wang K., Chen Y., Zhao Z., Feng M., Zhang S. (2019). Identification of Potential Core Genes and MiRNAs in Testicular Seminoma via Bioinformatics Analysis. Mol. Med. Rep..

[48] Bartel D.P. (2004). MicroRNAs: Genomics, Biogenesis, Mechanism, and Function. Cell.

[49] Chen X., Li X., Guo J., Zhang P., Zeng W. (2017). The Roles of MicroRNAs in Regulation of Mammalian Spermatogenesis. J. Anim. Sci. Biotechnol..

[50] Song Y., Kelava L., Zhang L., Kiss I. (2022). Microarray Data Analysis to Identify MiRNA Biomarkers and Construct the LncRNA-MiRNA-MRNA Network in Lung Adenocarcinoma. Medicine.

[51] Shi J., Gong L., Chen L., Luo J., Song G., Xu J., Lv Z., Tao H., Xia Y., Ye Z. (2019). MiR-618 Suppresses Metastasis in Gastric Cancer by Downregulating the Expression of TGF-Β2. Anat. Rec..

[52] Ivanovic R.F., Viana N.I., Morais D.R., Moura C., Silva I.A., Leite K.R., Pontes-Junior J., Nahas W.C., Srougi M., Reis S.T. (2018). MiR-618: Possible Control over TIMP-1 and Its Expression in Localized Prostate Cancer. BMC Cancer.

[53] Xie Z., Zhong C., Duan S. (2022). MiR-1269a and MiR-1269b: Emerging Carcinogenic Genes of the MiR-1269 Family. Front. Cell Dev. Biol..

[54] Castanhole-Nunes M.M.U., Tunissiolli N.M., Oliveira A.R.C.P., Mattos M.F., Galbiatti-Dias A.L.S., Kawasaki-Oyama R.S., Pavarino E.C., da Silva R.F., Goloni-Bertollo E.M. (2022). MiR-612, MiR-637, and MiR-874 Can Regulate VEGFA Expression in Hepatocellular Carcinoma Cell Lines. Genes.

[55] Tang J., Tao Z.H., Wen D., Wan J.L., Liu D.L., Zhang S., Cui J.F., Sun H.C., Wang L., Zhou J. (2014). MiR-612 Suppresses the Stemness of Liver Cancer via Wnt/β-Catenin Signaling. Biochem. Biophys. Res. Commun..

[56] Ibañez-Perez J., Díaz-Nuñez M., Clos-García M., Lainz L., Iglesias M., Díez-Zapirain M., Rabanal A., Bárcena L., González M., Lozano J.J. (2022). MicroRNA-Based Signatures Obtained from Endometrial Fluid Identify Implantative Endometrium. Hum. Reprod..

[57] Zhang Y., Zhang H., Yan L., Liang G., Zhu C., Wang Y., Ji S., He C., Sun J., Zhang J. (2023). Exosomal MicroRNAs in Tubal Fluid May Be Involved in Damage to Tubal Reproductive Function Associated with Tubal Endometriosis. Reprod. Biomed. Online.

[58] Zhou W., Lian Y., Jiang J., Wang L., Ren L., Li Y., Yan X., Chen Q. (2020). Differential Expression of MicroRNA in Exosomes Derived from Endometrial Stromal Cells of Women with Endometriosis-Associated Infertility. Reprod. Biomed. Online.

[59] Feng C., Shen J.M., Lv P.P., Jin M., Wang L.Q., Rao J.P., Feng L. (2018). Construction of Implantation Failure Related LncRNA-MRNA Network and Identification of LncRNA Biomarkers for Predicting Endometrial Receptivity. Int. J. Biol. Sci..

[60] Fu J., Qu R.G., Zhang Y.J., Gu R.H., Li X., Sun Y.J., Wang L., Sang Q., Sun X.X. (2018). Screening of MiRNAs in Human Follicular Fluid Reveals an Inverse Relationship Between MicroRNA-663b Expression and Blastocyst Formation. Reprod. Biomed. Online.

[61] Li Z., Zheng Z., Ruan J., Li Z., Zhuang X., Tzeng C.M. (2016). Integrated Analysis MiRNA and MRNA Profiling in Patients with Severe Oligozoospermia Reveals MiR-34c-3p Downregulates PLCXD3 Expression. Oncotarget.

[62] Heidary Z., Zaki-Dizaji M., Saliminejad K., Khorram Khorshid H.R. (2019). MicroRNA Profiling in Spermatozoa of Men with Unexplained Asthenozoospermia. Andrologia.

[63] Salas-Huetos A., Blanco J., Vidal F., Grossmann M., Pons M.C., Garrido N., Anton E. (2016). Spermatozoa from Normozoospermic Fertile and Infertile Individuals Convey a Distinct MiRNA Cargo. Andrology.

[64] Yao C., Yuan Q., Niu M., Fu H., Zhou F., Zhang W., Wang H., Wen L., Wu L., Li Z. (2017). Distinct Expression Profiles and Novel Targets of MicroRNAs in Human Spermatogonia, Pachytene Spermatocytes, and Round Spermatids between OA Patients and NOA Patients. Mol. Ther. Nucleic Acids.

[65] Piryaei F., Mozdarani H., Sadighi Gilani M.A., Rajender S., Finelli R., Darestanifarahani M., Sarli A., Mehta P., Agarwal A. (2023). Global Analysis in Nonobstructive Azoospermic Testis Identifies MiRNAs Critical to Spermatogenesis. Andrologia.

[66] Abu-Halima M., Khaizaran Z.A., Ayesh B.M., Fischer U., Khaizaran S.A., Al-Battah F., Hammadeh M., Keller A., Meese E. (2020). MicroRNAs in Combined Spent Culture Media and Sperm Are Associated with Embryo Quality and Pregnancy Outcome. Fertil. Steril..

[67] Noveski P., Popovska-Jankovic K., Kubelka-Sabit K., Filipovski V., Lazarevski S., Plaseski T., Plaseska-Karanfilska D. (2016). MicroRNA Expression Profiles in Testicular Biopsies of Patients with Impaired Spermatogenesis. Andrology.

[68] Rooda I., Kaselt B., Liivrand M., Smolander O.P., Salumets A., Velthut-Meikas A. (2021). Hsa-Mir-548 Family Expression in Human Reproductive Tissues. BMC Genom. Data.

[69] Lin F.J., Shen L., Jang C.W., Falnes P., Zhang Y. (2013). Ikbkap/Elp1 Deficiency Causes Male Infertility by Disrupting Meiotic Progression. PLOS Genet..

[70] Kaye E.G., Basavaraju K., Nelson G.M., Zomer H.D., Roy D., Joseph I.I., Rajabi-Toustani R., Qiao H., Adelman K., Reddi P.P. (2024). RNA Polymerase II Pausing Is Essential during Spermatogenesis for Appropriate Gene Expression and Completion of Meiosis. Nat. Commun..

[71] Kyrgiafini M.A., Sarafidou T., Mamuris Z. (2022). The Role of Long Noncoding RNAs on Male Infertility: A Systematic Review and In Silico Analysis. Biology.

[72] Nagirnaja L., Aston K.I., Conrad D.F. (2018). Genetic Intersection of Male Infertility and Cancer. Fertil. Steril..

[73] Da Ni F., Hao S.L., Yang W.X. (2019). Multiple Signaling Pathways in Sertoli Cells: Recent Findings in Spermatogenesis. Cell Death Dis..

[74] Lane D.P. (1992). Cancer. P53, Guardian of the Genome. Nature.

[75] Beumer T.L., Roepers-Gajadien H.L., Gademan I.S., Van Buul P.P.W., Gil-Gomez G., Rutgers D.H., De Rooij D.G. (1998). The Role of the Tumor Suppressor P53 in Spermatogenesis. Cell Death Differ..

[76] Raimondo S., Gentile T., Gentile M., Morelli A., Donnarumma F., Cuomo F., De Filippo S., Montano L. (2019). P53 Protein Evaluation on Spermatozoa DNA in Fertile and Infertile Males. J. Hum. Reprod. Sci..

[77] Fabian M.R., Sonenberg N., Filipowicz W. (2010). Regulation of MRNA Translation and Stability by MicroRNAs. Annu. Rev. Biochem..

[78] Gong J., Tong Y., Zhang H.M., Wang K., Hu T., Shan G., Sun J., Guo A.Y. (2012). Genome-Wide Identification of SNPs in MicroRNA Genes and the SNP Effects on MicroRNA Target Binding and Biogenesis. Hum. Mutat..

[79] Fu A., Hoffman A.E., Liu R., Jacobs D.I., Zheng T., Zhu Y. (2014). Targetome Profiling and Functional Genetics Implicate MiR-618 in Lymphomagenesis. Epigenetics.

[80] Feng X., Ji D., Liang C., Fan S. (2019). Does MiR-618 Rs2682818 Variant Affect Cancer Susceptibility? Evidence from 10 Case-Control Studies. Biosci. Rep..

[81] Radanova M., Mihaylova G., Mihaylova Z., Ivanova D., Tasinov O., Nazifova-Tasinova N., Pavlov P., Mirchev M., Conev N., Donev I. (2021). Circulating MiR-618 Has Prognostic Significance in Patients with Metastatic Colon Cancer. Curr. Oncol..

[82] Chen Y., Du M., Chen W., Zhu L., Wu C., Zhang Z., Wang M., Chu H., Gu D., Chen J. (2018). Polymorphism Rs2682818 in MiR-618 Is Associated with Colorectal Cancer Susceptibility in a Han Chinese Population. Cancer Med..

[83] Shao W., Xia H., Lan Q., Gu J., Huang H., Zheng F., Zheng Y. (2021). Polymorphism Rs2682818 Participates in the Progression of Colorectal Carcinoma via MiR-618-TIMP1 Regulatory Axis. Sci. Rep..

[84] Ozbayer C., Degirmenci I., Ustuner D., Ak G., Saydam F., Colak E., Gunes H.V., Metintas M. (2016). MiRSNPs of MiR1274 and MiR3202 Genes That Target MeCP2 and DNMT3b Are Associated with Lung Cancer Risk: A Study Conducted on MassARRAY Genotyping. J. Environ. Pathol. Toxicol. Oncol..

[85] Navabi A., Aznab M., Heydarpour F. (2022). The Association between MicroRNA Polymorphisms and the Risk of Childhood Acute Lymphoblastic Leukemia: A Meta-Analysis. Cancer Epidemiol..

[86] Pan H., Chen B., Wang J., Wang X., Hu P., Wu S., Liu Y., Xu Z., Zhang W., Wang B. (2016). The MiR-449b Polymorphism, Rs10061133 A>G, Is Associated with Premature Ovarian Insufficiency. Menopause.

[87] Rah H.C., Chung K.W., Ko K.H., Kim E.S., Kim J.O., Sakong J.H., Kim J.H., Lee W.S., Kim N.K. (2017). MiR-27a and MiR-449b Polymorphisms Associated with a Risk of Idiopathic Recurrent Pregnancy Loss. PLoS ONE.

[88] Kim J.O., Ahn E.H., Sakong J.H., An H.J., Park H.S., Kim Y.R., Lee J.R., Lee W.S., Kim N.K. (2020). Association of MiR-27aA>G, MiR-423C>a, MiR-449bA>G, and MiR-604A>G Polymorphisms with Risk of Recurrent Implantation Failure. Reprod. Sci..

[89] Castellini C., Cordeschi G., Tienforti D., Barbonetti A. (2024). Relationship between Male Aging and Semen Quality: A Retrospective Study on over 2500 Men. Arch. Gynecol. Obstet..

[90] Shi Z., Yu M., Guo T., Sui Y., Tian Z., Ni X., Chen X., Jiang M., Jiang J., Lu Y. (2024). MicroRNAs in Spermatogenesis Dysfunction and Male Infertility: Clinical Phenotypes, Mechanisms and Potential Diagnostic Biomarkers. Front. Endocrinol..

[91] Barbu M.G., Thompson D.C., Suciu N., Voinea S.C., Cretoiu D., Predescu D.V. (2021). The Roles of MicroRNAs in Male Infertility. Int. J. Mol. Sci..

[92] Spitzer T., Fujimoto V. (2013). Ethnic Differences in Assisted Reproductive Technologies Outcomes. Semin. Reprod. Med..

[93] Van Oosterhout C., Marcu D., Immler S. (2022). Accounting for the Genetic Load in Assisted Reproductive Technology. Clin. Transl. Med..

[94] Saint Pierre A., Génin E. (2014). How Important Are Rare Variants in Common Disease?. Brief. Funct. Genom..

