# Supplementary figures and images for: ΜicroRNA (miRNA) Variants in Male Infertility: Insights from Whole-Genome Sequencing
Source: Genes (Basel). 2024 Oct 29;15(11):1393. doi: 10.3390/genes15111393 (PMC11593656; doi:10.3390/genes15111393)

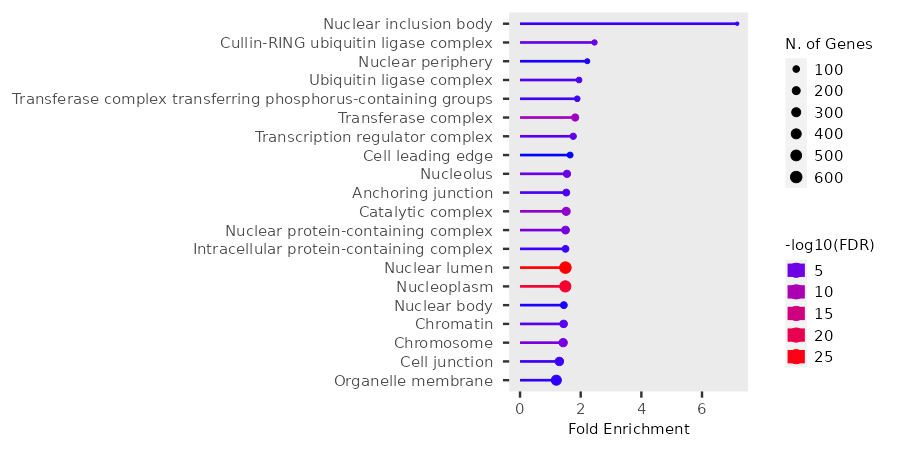

Supplement: Supplementary file 1 [file genes-15-01393-s001.zip › Figure S1.png]

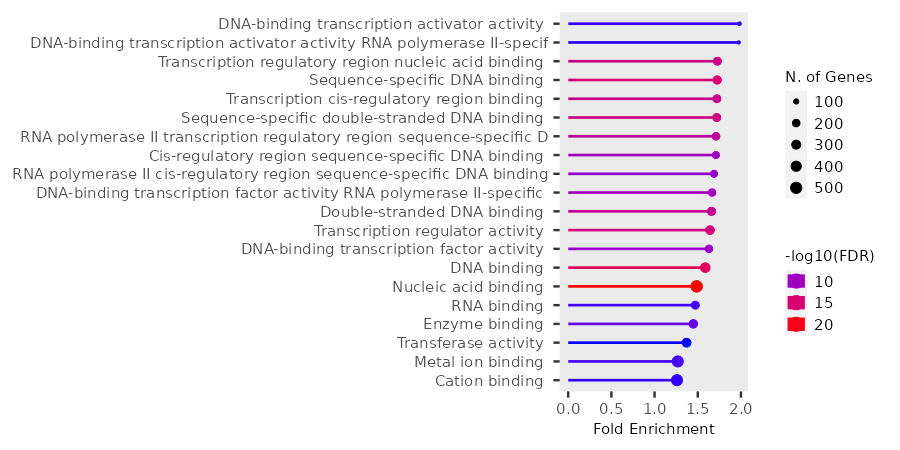

Supplement: Supplementary file 1 [file genes-15-01393-s001.zip › Figure S2.png]

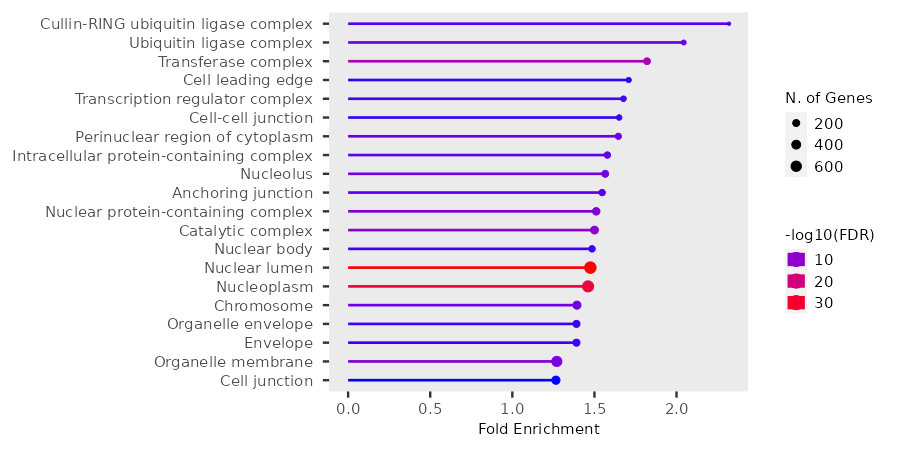

Supplement: Supplementary file 1 [file genes-15-01393-s001.zip › Figure S3.png]

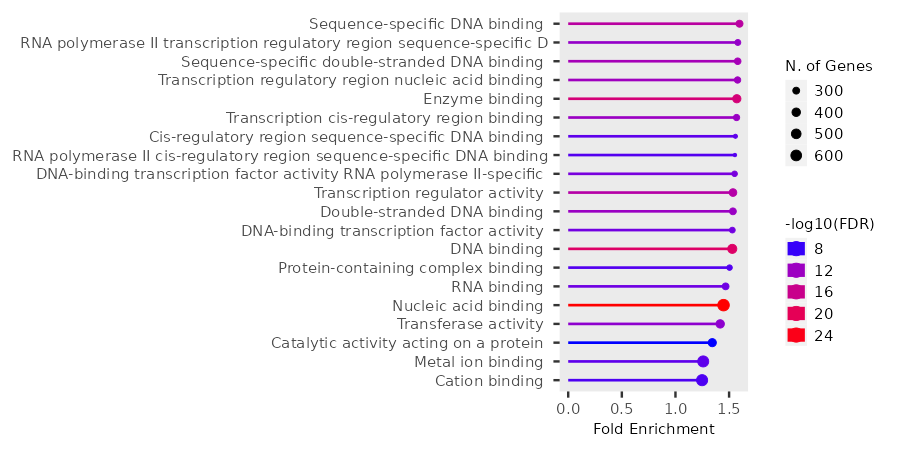

Supplement: Supplementary file 1 [file genes-15-01393-s001.zip › Figure S4.png]

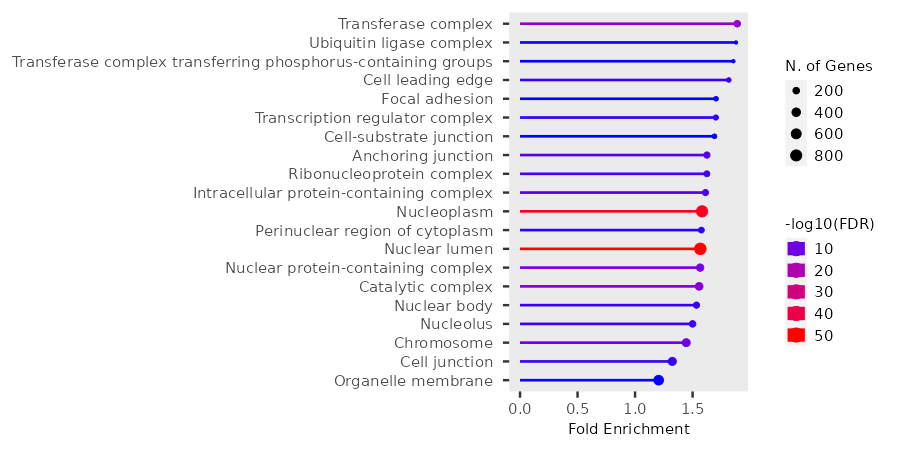

Supplement: Supplementary file 1 [file genes-15-01393-s001.zip › Figure S5.png]

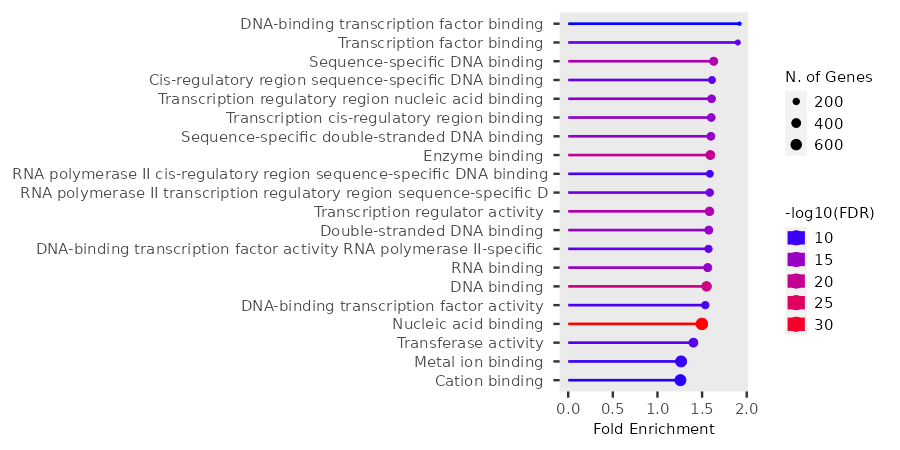

Supplement: Supplementary file 1 [file genes-15-01393-s001.zip › Figure S6.png]
